# Mixing ReaxFF parameters for transition metal oxides using force-matching method

**DOI:** 10.1007/s00894-021-04989-6

**Published:** 2021-12-14

**Authors:** Adam Włodarczyk, Mariusz Uchroński, Agata Podsiadły-Paszkowska, Joanna Irek, Bartłomiej M. Szyja

**Affiliations:** 1 Wrocław Centre for Networking and Supercomputing (WCSS), Wrocław University of Science and Technology, Wrocław, Poland; 2 Department of Fuels Chemistry and Technology, Wrocław University of Science and Technology, Gdańska 7/9, 50-344 Wrocław, Poland; 3 Present Address: Maria Curie-Skodowska University in Lublin, Pl. Marii Curie-Skłodowskiej 5, 20-031 Lublin, Poland

**Keywords:** Force-matching, Reactive forcefield, Hematite, Oxide nanoclusters

## Abstract

We present the development of the method for the refitting the ReaxFF parameters for a system consisting of the mixed transition metal oxides. We have tested several methods allowing to calculate the differences between the vectors of the forces acting on atoms obtained from the reference DFT simulation and the parameters-dependent ReaxFF. We conclude that the footrule method yields the best parameters among the investigated options. We then validate the parameters using the system consisting of the hematite supported (TiO_2_)_*n*_ clusters. The results indicate the refitted parameters allow to obtain acceptable geometries of the clusters upon MD simulation on the ReaxFF level, and despite the short timescale lead to the stable structures.

**Supplementary Information:**

The online version contains supplementary material available at 10.1007/s00894-021-04989-6.

## Introduction

Bridging the gaps between different scales is one of the most important challenges in simulations of chemical reactivity. The phenomena underlying the reactivity are related to the physical interactions between the electrons of the valence shell. That means that a proper description of the electronic structure of the reacting species is necessary for the evaluation of the activation energies, what in turn allows to simulate the changes in the atoms’ connectivity [1]. Practicality limits the simulations to relatively small systems, which can be handled by the electronic structure methods—such as Density Functional Theory. Despite the significant increase of the computational power in recent years, the size of the computational model in DFT simulation rarely exceeds 1000 atoms [2, 3].

In contrast to that, there are many classical potentials suitable to describe the interatomic forces; however, a key issue here is the static description of the chemical bonding, making use of the harmonic or Morse potential, what effectively prevents these methods to be used in the studies of chemical reactivity.

Among the currently available solutions for this issue, two are the most widely applied—Density Functional based Tight Binding [4–6] and ReaxFF [1, 7]. Both these methods rely on the parametrization, but the parameters are used in a different way. In DFTB, the parametrization is used in the Chebyshev polynomial expansion of the Hamiltonian and overlap matrix elements [4], what in (overly)simplified terms can be considered a DFT equivalent of the semi-empirical Hartree-Fock method [8]. ReaxFF takes a different approach, in which an extensive set of parameters and a complex energy expression is used to evaluate the bond orders between the atoms. Based on those, the interactions within the system are determined, similarly to classical forcefields.

The number of parameters needed for the ReaxFF description of the potential of the system is often in the order of hundreds, what makes the initial parametrization extremely difficult. Several methods have been investigated [9], including Monte Carlo–based simulated annealing algorithm [10, 11], genetic algorithms [12–15], covariance matrix adaptation [16–18] and machine learning [19]. However, due to the complexity of many-variable function optimization, the usual approach is to refit only some of the parameters to suit a particular system of interest. Even in this case, it has been reported that this task is often difficult to algorithmize, and methods involving heuristics outperform traditional parametrizations [20].

Virtually always the parameters are being fitted with the energetic criterion as the target function. In this approach, the target is the minimum difference between the energy of the system obtained with given parameters and the reference energies, often taken from higher level calculations. This allows to reproduce the dissociation energy curve directly and is often a standard procedure in parameters optimization.

In this work, we take a different approach—a force-matching, which was first proposed by Ercolessi and Adams [21]. In this approach, the potential is fitted to the atomic forces obtained from the higher level calculations. This implies that a target function cannot be just one scalar value for the given geometry of the system being investigated, but rather a set of values representing the force vectors acting on each atom. In the case of a 3D system, as in chemical simulations, this results in matching 3*N* values—where *N* is the number of atoms—and in essence leads to a rather complex target function. In this work, we present a comparison of selected methods for the calculation of the differences between the forces acting on the atoms from ReaxFF and DFT.

Hence, the aim of our work is twofold: (1) the optimization of the set of parameters for our specific system of interest, and (2) the development of the optimization procedure, which can be used to optimize the parameters for other systems. We need to stress out that this procedure might not, and—most likely—will not be applicable in all cases, and we do not claim such universality. However, the comparison presented by us can serve such a purpose, and we believe other researchers will find it useful to optimize the parameters for their systems of interest.

## Force matching procedure

The main idea behind the force matching procedure presented in this work is based on the island model, which consists of multiple local optimizing procedures and once in a while exchanging the results between each island [22]. In this particular procedure, the modelled island is a given geometry (a snapshot from a trajectory) set of ReaxFF parameters and reference DFT forces. The goal is to find a universal set of parameters which will give a sufficient result, in terms of some target function, for all geometries in the trajectory.

The procedure assumes that the differences in forces obtained using the generated parameters set are computed, and compared with the reference DFT forces. In this work, we have used VASP [23–26] to provide the reference values of the forces. The forces have been calculated (and compared) for each of the atom in the model. The force vectors have been calculated for each atom with respect to full environment of the particular atom. The trajectory was generated starting from an optimized geometry, with the temperature of 350 K controlled by a Nose thermostat. The timestep was set to 1 fs, and the total time of simulation was set to 1 ps (1000 steps). From the full trajectory we have randomly chosen 10 frames, which served the basis for each island.

With a starting set of parameters as a gulp configuration file, the subset of selected parameters was modified in order to obtain a better result in terms of an objective function. Considering the island model, each of those ten trajectories is an island for which the objective function is computed in each optimization iteration and those results are summed up to give a result for the given iteration. If the result is better, then the considered set of tweaked parameters for this particular iteration is treated as the best result and from now on the further tweaked set is compared to this one. The optimization process assumes 2000 iterations and for each of them there is a random selection of one to 16 (the number of parameters to consider is also randomly taken) to tweak out of the 31 selected parameters to be modified. For each of the selected parameters, there is a random component to be added which is different up to ±,5% from the considered parameter value. Such a modified subset of parameters along with the constant ones is applied for each trajectory to obtain an objective.

The parametrization was focused on the modification of the most relevant parameters from the point of view of the investigated model—i.e. those for Ti atoms, O atoms and Ti-O bonds. The justification of this selection is discussed in the SI.

The modification of selected parameters is a random change of maximum ± 5% for a randomly chosen parameter, with bounds set as ± 30% of the initial value of this parameter. In other words, each of the indicated parameters has its initial value which will be remembered across all of the optimization steps and its values of 70% and 130% are treated as lower and upper bound, respectively. These initial parameters can be used directly from the available parameters sets, i.e. from the literature. A single parameter change is uniformly distributed between 95 and 105% of the value of the parameter in given iteration. The choice of a parameter to be modified is performed with uniform distribution draw as well. For each trajectory a separate modification of the parameters is made, so the global comparison, i.e. for each geometry in the trajectory, is necessary. Such comparison, i.e. the object function, can be executed in several manners. We investigated the following metrics to measure the difference between reference forces and those resulting from tweaked parameters:

- *Euclidean distance* – a standard distance metric in euclidean space, i.e. the Minkowski distance with *p* = 2. Equation 1, where *n* denotes size of vectors *X* and *Y*, *x*_*i*_ and *y*_*i*_ denote the *i*^*th*^ component of the respective vectors.
  1 $$  D_{ed}(X, Y) = \sqrt {{\sum}_{i=1}^{n}{{\left | x_{i} - y_{i} \right |}^{2}}} $$
- *Cosine similarity* – a quotient of the dot product of vectors *X* and *Y*, and the product of norms of those vectors. Equation 2.
  2 $$  D_{cs}(X, Y) = \frac{X\cdot Y} {\left \| X \right \| \left \| Y \right \|} $$
- *Norms difference* – an absolute value of the difference of norms of vectors *X* and *Y*. Equation 3.
  3 $$  D_{nd}(X, Y) = \left | \left \| X \right \| - \left \| Y \right \| \right | $$
- *PCC* – a Pearson correlation coefficient representing the correlation between vectors *X* and *Y*. Equation 4, where *n* denotes size of both vectors, $\overline {x}, \overline {y}$ denote means of the respective vectors, and *x*_*i*_ and *y*_*i*_ denote the *i* th component of the respective vectors.
  4 $$  D_{pcc}(X, Y) = \frac{{\sum}_{i=1}^{n} (x_{i} - \overline{x}) (y_{i}- \overline{y})} {\sqrt{{\sum}_{i=1}^{n} (x_{i} -\overline{x})^{2} {\sum}_{i=1}^{n} (y_{i} -{\overline{y})^{2}}}} $$
- *Footrule* – the Spearman’s footrule which is a measure of disarray between two vectors. Equation 5, where *n* denotes size of vectors *X* and *Y*, and *x*_*i*_ and *y*_*i*_ denote the *i* th component of the respective vectors
  5 $$  D_{f}(X, Y) = \sum\limits_{i=1}^{n}\left | x_{i} - y_{i} \right | $$

The pseudocode of the parameters optimization procedure is presented in Algorithm 1. The most important part of input for the procedure consists of the initial set of ReaxFF parameters in the GULP [27, 28] *lib* format, with chosen and marked parameters to be optimized. For those parameters the upper and lower bounds are computed (70% and 130% of each initial parameters value as the lower and upper bound, as described above). Also, the set of geometries with the reference forces was provided. This set of geometries and corresponding forces, for which the force matching would be performed, was considered a training set. The essential paralleled part of the algorithm are GULP computations of forces for each given geometry, with use of a modified set of parameters. Since those computations are executed separately, this is an *embarrassingly parallel* problem. 
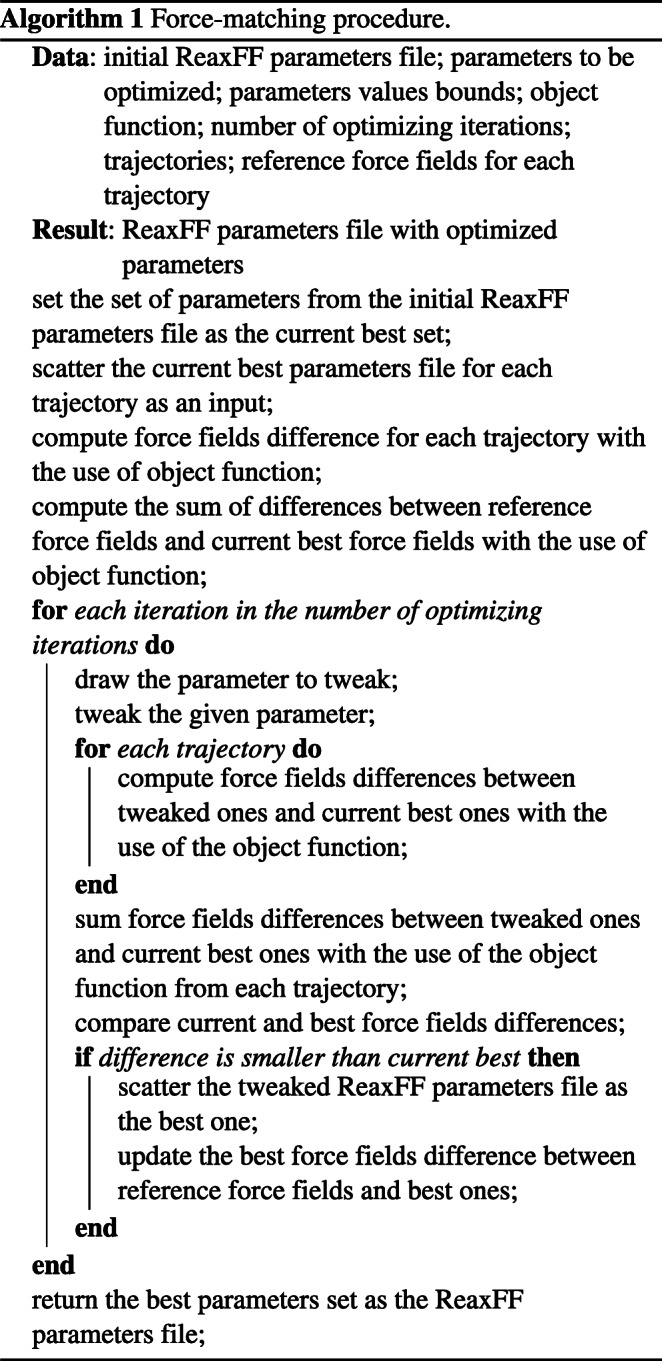


For the implementation, we have used a Python programming language with the use of mpi4py library to parallelize and distribute computation across multiple processors by MPI interface. The ReaxFF computations were performed by wrapped GULP software [27]. The custom software was used to optimize, tweak the selected parameters and to provide several minor functionalities. Computations were performed on a cluster of 16 Intel Xeon E5-2683 CPUs with 16 GB of RAM and CentOS 7 operating system. The force matching procedure was performed for 10 different trajectories with optimization of six parameters for 2000 iterations.

Last but not least, the verification of the optimized parameters have been done indirectly by means of the comparison of the dissociation energy curves. The comparison of bond lengths and angles brings very little to the results, as these are the parameters of the ReaxFF, and varying them in order to directly compare them after MD run is in our opinion pointless. Therefore, in this work we rely on the comparison of the dissociation energy curves, as they provide indirect verification of the bonding characteristics and reproduction of the shape of the energy curve is the most important from the MD point of view, where energy gradients determine the forces acting on atoms. Furthermore, we have carried out short (3 ps) ReaxFF MD simulations in order to evaluate stability of the systems.

## Model system: TiO_2_ cluster on the hematite surface

In the case of (TiO_2_)_*n*_ clusters on hematite we in fact considered several different systems—a clean hematite (110) surface, free-standing TiO_2_ clusters of different size and shape and finally we tested two clusters on the surface.

For the iron oxides and oxohydroxide systems, there are two sets of ReaxFF parameters available in the literature—one of them is for the bulk materials and the other for the surface-water interface [29]. Here we consider a different system—we use a slab consisting of 5 monolayers of the hematite (110) 2 × 2 surface with a vacuum layer of the thickness of approximately 20 Å. Prior to the optimization procedure, we have tested both sets of parameters, taking into account that a set of parameters should be transferable into similar systems.

The above parameters were matched to the data from DFT, which have been performed using the VASP package [25, 26]. The exchange-correlation functional was used in the Perdew-Burke-Ernzerhof [30] form. The energy cutoff was set to 500 eV and the electron–ion interactions were described by the projector augmented waves (PAW) method [31, 32]. Due to the large unit cell, the energy was calculated only at the Γ-point.

The characteristics of the system obtained from the simulation with the literature ReaxFF parameters were not comparable with the one from DFT in two respects—the Fe-O distances and surface stability. *α*-Fe_2_*O*_3_ consists of the octahedra with oxygen atoms in the vertices and an Fe atom occupying the site near the geometrical center [33]. As the Fe atom is slightly shifted from the octahedron center the Fe-O bonds have two different lengths. This was confirmed in our DFT calculations [34], but the system obtained with the given ReaxFF parameters did not exhibit this feature, i.e. all Fe-O bonds had the same length.

The other problem is the surface instability, which may result from the fact that our calculations were carried out at temperatures much higher than 5K, for which FF parameters were fitted. In the paper of Aryanpour et al. [29] the goal was to eliminate the kinetic part making its effect on the cell volume negligible. This approach can be justified as it significantly simplifies calculations and it works properly for the bulk systems. However, such parameters are not transferable to a slab system. At higher temperatures, the strong fluctuations of the whole layers of atoms with respect to the *z*-coordinate have been observed—showing alternating contraction and expansion steps. This is likely a result of a too steep energy curve away from the energy minimum, which leads to this unrealistic behaviour.

Another evidence for insufficient transferability of these parameters is an incorrect description of particular atoms of the structure. In the temperature below RT, approximately 200 K one of the Fe atoms of the topmost layer migrated from its initial position and it remained on the surface until the end of the simulation. That was observed even though in the MD calculations we increased the temperature gradually from 0 or 100 to 300 K. This way, we avoided the unreasonable growth of the temperature in the beginning of the calculations, which could have caused this effect. Nevertheless, at the temperature of approximately 300 K, another Fe atom also migrated from the surface, making the surface clearly unstable at room temperature.

**Fig. 1:**
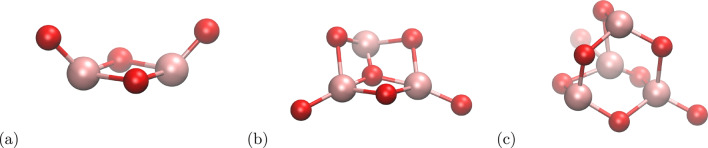
Structures of the (TiO_2_)_*n*_ clusters. (a) *n* = 2 – “2b”, (b) *n* = 3 – “3a”, (c) *n* = 4 – “4a”. Titanium and oxygen are shown in pink and red colour, respectively. The names of the clusters are adopted from the work of Qu et al. [36]

The second component of this study was TiO_2_ clusters. We used the parameters fitted for the system consisted of TiO_2_ nanoparticles with water, methanol, and formic acid [35] and the most energetically favourable shapes of different sizes of the clusters [36]. The parameters were fitted for the system at temperature of 300 K; thus, we did not expect any problem of clusters stability. Almost all of the tested clusters, i.e. with *n*-number ranged from 2 to 4 were stable in the calculations. Only the cluster in geometry 2c transformed into the geometry 2a, but it can be considered a transition to a lower energy state, as in the simulation temperature it was possible to overcome the activation energy barrier. The structures of the clusters adopted from the work of Qu et al. [36] are shown in Fig. 1.

The aim of these calculations was to combine both systems putting the TiO_2_ clusters on the hematite surface. This system is quite specific and as such it is difficult to study experimentally with sufficient details to provide the atomistic description of its structure, and we are not aware of any experimental literature reports on this matter. The combination of atom types is not available in the literature for the ReaxFF simulations either; therefore, we have combined both sets of parameters, i.e. for Fe-O and Ti-O systems, with subsequent refitting of the dissociation energies and the bond lengths. The oxygen parameters were essentially the same in both sets. Moreover, we do not expect the possibility of Fe-Ti bond formation, and we assume that Fe and Ti can bind only through O bridges. Clearly, in this case, there are many parameters that should be taken into account for refitting, i.e. bond lengths, dissociation energies, angles and dihedral angles. Therefore, a careful selection of the parameter set was carried out.

**Fig. 2:**
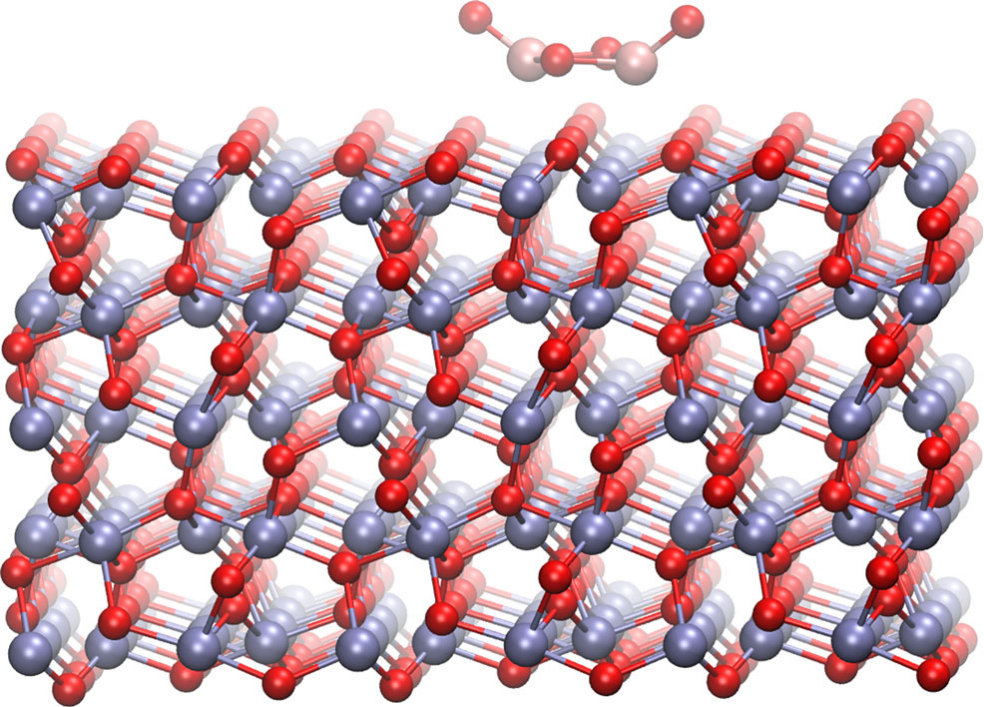
System used as a training set. Iron, titanium and oxygen atoms are shown in blue, pink and red color, respectively

The system we used for the tests was the highly symmetrical (TiO_2_)_2_ cluster, i.e. the geometry 2b of the work of Qu et al. [36], positioned arbitrarily on the surface in the distance of 1.7 Å–see Fig. 2. For this cluster, we have performed 3 ps long MD calculations in 300 K on DFT level. Subsequently, we have selected 10 frames separated by 0.3 ps out of the resulting trajectory, and for these geometries we have calculated forces from the DFT. The ReaxFF parameters were fitted so the forces from ReaxFF were as close as possible to the DFT results, using the procedure described in “Force matching procedure”.

**Fig. 3:**
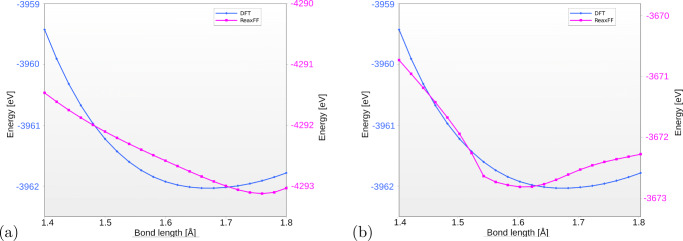
The energy curve for dissociation one of the O atoms from the 2b cluster on the surface. Results obtained for the parameters from literature [29] (a), and ReaxFF parameters obtained by minimization of the norm of the difference of force vectors (b). The ReaxFF results are denoted by purple lines; the blue lines are the DFT results

To test the accuracy, we computed the energy curve for dissociation of one of the oxygen atoms of the Ti-O-Fe group. It has to be stressed again that the energy was never taken as a reference in our procedure, and it is strictly the forces that have been matched. Thus, the comparison of the energy curves is an indirect result of the force matching procedure. The results are shown in Fig. 3.

Clearly, the shape of the energy curve obtained in DFT calculations is not reproduced well by any of the parameters sets. DFT results are characterized by a smooth minimum, while ReaxFF calculations result in more sharp curves for both sets of parameters. This is consistent with our previous observations, where the alternating contractions/expansions have been observed. The key factor in the new set of parameters is that the energy minimum is now shifted to a higher value—from 1.6 to 1.75 Åwhich overestimates the value from the DFT. These results show clearly that the method of parameters refitting is not perfect. The tests confirmed these observations: for some clusters it gave better results—the cluster interacting with the surface became more stable, for the others we have not observed any improvement.

**Fig. 4:**
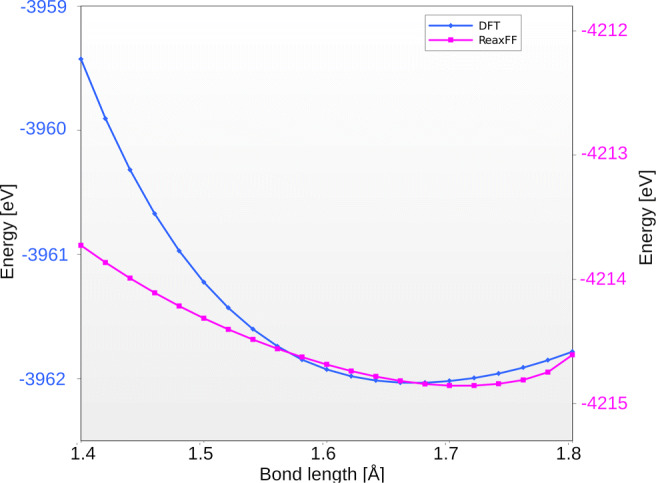
Dissociation energy curve for footrule-method fitted parameters. The ReaxFF results are denoted by purple lines, the blue lines are the DFT results

Searching for a better working parameters set, we tested other parameters matching methods as described in “Force matching procedure”. The best results we have obtained for the footrule method—Fig. 4.

The shape of the energy curve for this method was the most similar to the DFT results and we achieved very good agreement with the location of the minimum energy curve compared to the results from DFT. It needs to be pointed out that the depth of the minimum (dissociation energy) is not matching well the DFT, especially for short interatomic distances. This, however, seems to have little adverse effects on the trajectories generated using this parameter set.

The obtained set of parameters served as the base for the series of the MD simulations on the ReaxFF level in order to validate the results. For each of the investigated geometries of the (TiO_2_)_*n*_ clusters, a series of MD simulations have been carried out, with the initial geometry consisting of each of the clusters and the hematite surface. The MD simulations have been carried out using the same method, as described in “Force matching procedure” —the temperature was set to 100 K for the first 100 steps of the run, and then gradually increased to 300 K over the subsequent 200 steps. In order to control the temperature, the Nosé–Hoover thermostat has been used. Total simulation time was set to 3 ps (3000 steps) as all systems have reached the stability by the end of these simulations. Extending the runs to 30 ps (30 000 steps) did not significantly change the final geometry. Only selected trajectories are discussed here.

**Fig. 5:**
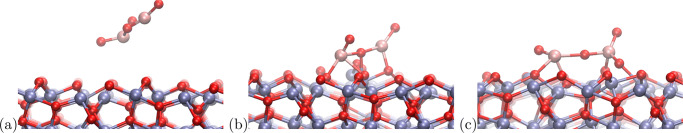
Snapshots from the MD simulation of the (TiO_2_)_2_ cluster 2b. (a) frame 620, (b) frame 1263, (c) frame 3000

Figure 5 shows the snapshots from the simulation of the 2b system, consisting of (TiO_2_)_2_ cluster and the hematite surface. The initial configuration is shown in Fig. 2. Only insignificant conformational changes are taking place in the first phase of the simulation, when the cluster interacts weakly with the surface. The only significant change that is visible in the trajectory in the first picosecond is the rotation of the cluster, where the cluster initially oriented parallel to the surface, reorients with respect to the surface with one of the Ti atoms. This is shown in Fig. 5(a).

Subsequently, at approximately 1.2 ps, the cluster forms the bonds with the surface, and one of the Ti-O-Ti bridges is cleaved. The oxygen atom from this bridge binds to the hematite surface, what causes one of the Fe atoms to be pulled out from the surface. This is shown in Fig. 5(b). This configuration remains stable until the end of the simulation; however, the Fe atom migrates back to its original position—see Fig. 5(c).

**Fig. 6:**
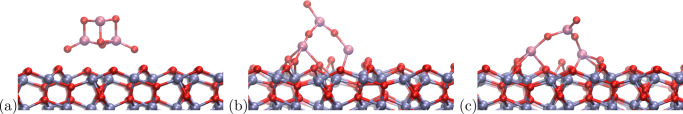
Snapshots from the MD simulation of the (TiO_2_)_3_ cluster 3a. (a) frame 1, (b) frame 845, (c) frame 3000

Figure 6(a) shows the initial configuration of the (TiO_2_)_3_ cluster. The cluster is initially oriented similarly to the 2b one, with one additional TiO_2_ unit located further above the surface. One of the oxygen atoms binds to three Ti atoms, and—as it is located close to the hematite surface—it should be considered overcoordinated.

Indeed, the bonds of this O atom are the ones to be cleaved first, and in 0.8 ps of the MD run the cluster changes the shape significantly. Two of the Ti atoms that were initially located close to the surface, bind to the oxygen atoms of the hematite, and the top Ti atom remains bound to the other Ti atoms, however, only via single Ti-O-Ti bridges. The previously overcoordinated oxygen atom dissociates from the cluster and binds to the Fe atoms of the hematite, forming a bridging site. This is shown in Fig. 6(b).

This structure remains stable until the end of the MD run; however, the top Ti atom migrates closer to the hematite surface, away from the dissociated oxygen atom. Interestingly, this Ti atom remains undercoordinated, as it forms bonds with one lone oxygen and two bridging oxygens. In our view, this should be considered an artifact, most likely coming from the too shallow curvature of the dissociation energy shown in Fig. 4. Overcoordinated oxygen atom dissociates too quickly from the cluster, and migrates too far away from the Ti atom, to allow for the bond formation.

**Fig. 7:**
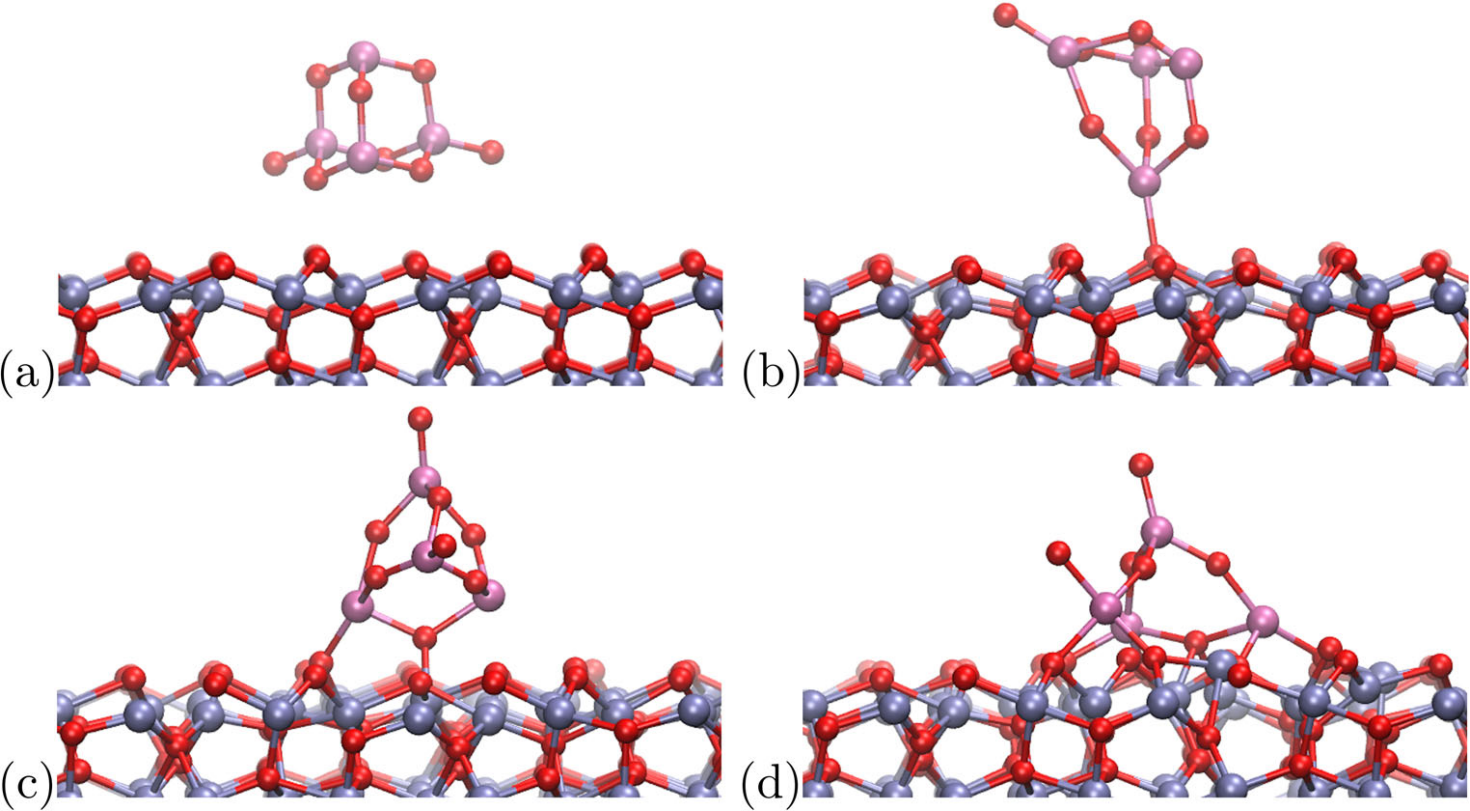
Snapshots from the MD simulation of the (TiO_2_)_4_ cluster 4a. (a) frame 1, (b) frame 910, (c) frame 1665, (d) frame 3000

The 4a cluster shown in Fig. 7 maintains its shape throughout the most of the MD run. In the initial phase of the simulation, the cluster rotates, and forms a bond with the hematite oxygen atom. This allows the Ti atom from the cluster to fill up the fourth coordination spot. This, however, does not lead to any other bond cleavage, and the cluster is only slightly distorted. This is shown in Fig. 7(b).

The formation of this bond leads to the immobilization of the cluster on the surface and brings the cluster closer to the hematite surface, what in turn leads to the formation of the bond between the oxygen atom from the cluster and the iron atom from the surface. This is shown in Fig. 7(c). In the final phase, one of the Ti-O-Ti bridges is cleaved and the cluster forms direct Ti-O-Fe connections. All four Ti atoms maintain 4-fold coordination, with the top Ti atom being the only one that is not bound directly to the surface.

Similarly to the case of the 2b cluster, one of the Fe atoms pops out of its crystallographic position in the surface, due to the interactions with the cluster. This is shown in Fig. 7(d). However, the distortion of the hematite surface is minor.

## Conclusions

This work describes the procedure of the force-matching applied to fitting the ReaxFF parameters for the *TiO*2 cluster on a hematite (110) surface. As the starting point, we have used the parameters available in the literature. These parameters could not be used directly, because the MD simulations revealed poor stability of the cluster and incorrect behaviour of the hematite surface.

We have tested different algorithms for the force-matching procedure, with respect to the reference forces provided by DFT simulations. We have tested the quality of the resulting parameters by comparing the dissociation energy curves. The footrule method provided the most smooth energy curve and the smallest difference with respect to the equilibrium bond lengths.

The final validation of the parameters has been accomplished by means of the MD simulations using ReaxFF potential and fitted parameters. We have observed significant improvement with respect to the overall stability of the system. The geometry of the cluster has been mostly maintained throughout all the simulations; however, we have observed the dissociations of the Ti-O-Ti bridges. This could have been caused by the interactions with the hematite surface; however, the insufficient quality of the parameters cannot be yet excluded. One has to remember that the dissociation energy curve obtained from the ReaxFF parameters is shallow, and as such it promotes the cleavage of Ti-O bonds.

The depth of the dissociation energy curve, however, was only a result of the force-matching, and at no point it was used as a reference to obtain a set of ReaxFF parameters. Despite that, the cluster geometries obtained from the validation MD runs, show the characteristics of stable structures. While the further fitting would probably needed to achieve the chemical accuracy of the activation energies, the obtained geometries can well serve as the starting point for DFT investigations.

## Electronic supplementary material

Below is the link to the electronic supplementary material.

(PDF 968 KB)

## Data Availability

Please contact the authors with respect to the availability of the code used for force-matching.

## References

[1] Senftle TP, Hong S, Islam MM, Kylasa SB, Zheng Y, Shin YK, Junkermeier C, Engel-Herbert R, Janik MJ, Aktulga HM, Verstraelen T, Grama A, Van Duin ACT (2016) The ReaxFF reactive force-field: development, applications and future directions. npj Computational Materials, 2(1)

[2] Soler JM, Artacho E, Gale JD, Garcł́a A, Junquera J, Ordejón P, Sánchez-Portal D (2002). The SIESTA method forab initioorder-nmaterials simulation. J Phys Condens Matter.

[3] García A, Papior N, Akhtar A, Artacho E, Blum V, Bosoni E, Brandimarte P, Brandbyge M, Cerdá JI, Corsetti F, Cuadrado R, Dikan V, Ferrer J, Gale J, García-Fernández P, García-Suárez VM, García S, Huhs G, Illera S, Korytár R, Koval P, Lebedeva I, Lin L, López-Tarifa P, ayo SG, Mohr S, Ordejón P, Postnikov A, Pouillon Y, Pruneda M, Robles R, Sánchez-Portal D, Soler JM, Ullah R, Wen-zhe Yu V, Junquera J (2020). Siesta: recent developments and applications. J Chem Phys.

[4] Porezag D, Frauenheim TH, Köhler TH, Seifert G, Kaschner R (1995). Construction of tight-binding-like potentials on the basis of density-functional theory application to carbon. Phys Rev B.

[5] Elstner M, Porezag D, Jungnickel G, Elsner J, Haugk M, Frauenheim TH, Suhai S, Seifert G (1998). Self-consistent-charge density-functional tight-binding method for simulations of complex materials properties. Phys Rev B.

[6] Seifert G, Porezag D, Frauenheim TH (1996). Calculations of molecules, clusters, and solids with a simplified LCAO-DFT-LDA scheme. Int J Quantum Chem.

[7] van Duin ACT, Dasgupta S, Lorant F, Goddard WA (2001). ReaxFF: a reactive force field for hydrocarbons. J Phys Chem A.

[8] Cramer C (2004). Essentials of computational chemistry : theories and models.

[9] Shchygol G, Yakovlev A, Trnka T, van Duin ACT, Verstraelen T (2019). ReaxFF parameter optimization with monte-carlo and evolutionary algorithms, Guidelines and insights. J Chem Theory Comput.

[10] Iype E, Hütter M, Jansen APJ, Nedea SV, Rindt CCM (2013). Parameterization of a reactive force field using a monte carlo algorithm. J Comput Chem.

[11] Kirkpatrick S, Gelatt CD, Vecchi MP (1983). Optimization by simulated annealing. Science.

[12] Müller J, Hartke B (2016). reaxFF reactive force field for disulfide mechanochemistry, fitted to multireference ab initio data. Journal of Chemical Theory and Computation.

[13] Dittner M, Müller J, Aktulga HM, Hartke B (2015). Efficient global optimization of reactive force-field parameters. J Comput Chem.

[14] Pahari P, Chaturvedi S (2011). Determination of best-fit potential parameters for a reactive force field using a genetic algorithm. J Mol Model.

[15] Jaramillo-Botero A, Naserifar S, Goddard WA (2014). General multiobjective force field optimization framework, with application to reactive force fields for silicon carbide. J Chem Theory Comput.

[16] Hansen N, Kern S (2004) Evaluating the cma evolution strategy on multimodal test functions. In: Yao Xin, Burke EK, Lozano JA, Smith J, Julián Merelo-Guervós J, Bullinaria JA, Rowe JE, Tiño P, Kabán A, Schwefel H-P (eds) Parallel Problem Solving from Nature - PPSN VIII. Springer, Berlin, pp 282–291

[17] Dürholt JP, Fraux G, Coudert F-X, Schmid R (2019). Ab initio derived force fields for zeolitic imidazolate frameworks MOF-FF for ZIfs. J Chem Theory Comput.

[18] Trnka T, Tvaroška I, Koča J (2017). Automated training of reaxFF reactive force fields for energetics of enzymatic reactions. J Chem Theory Comput.

[19] Sengul MY, Song Y, Nayir N, Gao Y, Hung Y, Dasgupta T, van Duin ACT (2021) INDEEDopt: a deep learning-based ReaxFF parameterization framework. 7(1)

[20] Barcaro G, Monti S, Sementa L, Carravetta V (2017). Parametrization of a reactive force field (reaxFF) for molecular dynamics simulations of si nanoparticles. J Chem Theory Comput.

[21] Ercolessi F, Adams JB (1994). Interatomic potentials from first-principles calculations: the force-matching method. Europhysics Letters (EPL),.

[22] Whitley D, Rana S, Heckendorn RB (1997) Island model genetic algorithms and linearly separable problems. In: Corne D, Shapiro JL (eds) Evolutionary Computing. Springer, Berlin, pp 109–125

[23] Kresse G, Hafner J (1993). Ab initiomolecular dynamics for liquid metals. Phys Rev B.

[24] Kresse G, Hafner J (1994). Ab initiomolecular-dynamics simulation of the liquid-metal–amorphous-semiconductor transition in germanium. Phys Rev B.

[25] Kresse G, Furthmüller J (1996). Efficiency of ab-initio total energy calculations for metals and semiconductors using a plane-wave basis set. Comput Mater Sci.

[26] Kresse G, Furthmüller J (1996). Efficient iterative schemes forab initiototal-energy calculations using a plane-wave basis set. Phys Rev B.

[27] Gale JD (1997). GULP: A computer program for the symmetry-adapted simulation of solids. Journal of the Chemical Society Faraday Trans.

[28] Gale JD, Raiteri P, van Duin ACT (2011). A reactive force field for aqueous-calcium carbonate systems. Phys Chem Chem Phys.

[29] Aryanpour M, van Duin ACT, Kubicki JD (2010). Development of a reactive force field for iron-oxyhydroxide systems. J Phys Chem A.

[30] Perdew JP, Burke K, Ernzerhof M (1996). Generalized gradient approximation made simple. Phys Rev Lett.

[31] Blöchl PE (1994). Projector augmented-wave method. Phys Rev B.

[32] Kresse G, Joubert D (1999). From ultrasoft pseudopotentials to the projector augmented-wave method. Phys Rev B.

[33] Fasiska EJ (1967). Structural aspects of the oxides and oxyhydrates of iron. Corros Sci.

[34] Podsiadły-Paszkowska A, Tranca I, Szyja BM (2019). Tuning the hematite (110) surface properties to enhance its efficiency in photoelectrochemistry. J Phys Chem C.

[35] Kim SY, van Duin ACT, Kubicki JD (2012). Molecular dynamics simulations of the interactions between TiO2 nanoparticles and water with na+ and cl-, methanol, and formic acid using a reactive force field. J Mater Res.

[36] Qu Z-W, Kroes G-J (2006). Theoretical study of the electronic structure and stability of titanium dioxide clusters (tio2)n with n = 1-9. J Phys Chem B.

