# Supplementary material for: Mixing ReaxFF parameters for transition metal oxides using force-matching method
Source: J Mol Model. 2021 Dec 14;28(1):8. doi: 10.1007/s00894-021-04989-6 (PMC8671285; doi:10.1007/s00894-021-04989-6)
Supplement: Supplementary file 1 — (PDF 968 KB) [file 894_2021_4989_MOESM1_ESM.pdf]

Mixing ReaxFF parameters for transition metal oxides using force-matching – Supporting Information

Adam Włodarczyk[1], Mariusz Uchroński[1], Agata Podsiadły-Paszkowska[2], Joanna Irek[2], and Bartłomiej M. Szyja*[2]

[1]Wrocław Centre for Networking and Supercomputing (WCSS), Wrocław University of Science and Technology
[2]Department of Fuels Chemistry and Technology, Wrocław University of Science and Technology, Gdańska 7/9, 50-344 Wrocław, Poland
b.m.szyja@pwr.edu.pl

November 8, 2021

1 ReaxFF parameters in GULP lib format

The listing contains the complete set of parameters used in the study. The lines containing "modify" keyword denote the lines with the parameters for tweaking, and the numbers after the keyword denote which parameter in the particular line is to be tweaked.

```
#
#  ReaxFF force field
#  ------------------

reaxFFvdwcutoff      10.0000
reaxFFqcutoff        10.0000
reaxFFtol      0.001 0.001 0.000001 0.01 7.5 0.000000001
#
#  Species independent parameters
#
reaxff0_bond        50.000000     9.546900
reaxff0_over        50.000000     0.699100     1.058800    12.117600    13.305600
reaxff0_valence     33.866700     1.851200     1.056300     2.038400
reaxff0_penalty      6.929000     0.398900     3.995400
reaxff0_torsion      5.779600    10.000000     1.948700     2.164500
reaxff0_vdw          1.559100
reaxff0_lonepair     6.089100
#
#  Species
#
species
O  core   0.000
Fe core   0.000
Ti core   0.000
#
#  Species parameters
#
reaxff1_radii
O  core   1.2450   1.0548   0.9049
Fe core   1.9506   2.0500  -1.2000
Ti core   2.0254   0.1000  -1.0000
reaxff1_valence
O  core   2.0000   4.0000   6.0000   4.0000
Fe core   3.0000   6.0000   3.0000   3.0000
Ti core   4.0000  19.9797   4.0000   4.0000
```

```
reaxff1_over
O  core   0.7640   3.5027   0.0021  -3.5500
Fe core   1.0000  30.0000   0.0000 -16.2040
Ti core   0.5782  19.9797   0.0064 -15.0000
reaxff1_under kcal
O  core  37.5000
Fe core   0.0000
Ti core   0.1000
reaxff1_lonepair kcal
O  core   2.0000   0.4056
Fe core   0.0000   0.0000
Ti core   0.0000   0.0000
reaxff1_angle
O  core   2.9000   2.9225
Fe core   2.7917   2.5791
Ti core   1.5000   2.2632
reaxff1_morse kcal
O  core   9.7300   0.1000   2.3890  13.8449
Fe core  11.0534   0.1274   2.0308   2.2637
Ti core  12.7041   0.1574   2.2105  16.6482
#
#  Element parameters
#
reaxff_chi
O  core   8.5000
Fe core   1.2457
Ti core  -4.6395
reaxff_mu
O  core   8.3122
Fe core   7.3021
Ti core   7.7218
reaxff_gamma
O  core   1.0898
Fe core   0.7264
Ti core   0.4098
#
#  Bond parameters
#
reaxff2_bo over bo13
O  core O  core  -0.1225   5.5000  -0.1055   9.0000  -0.1000  29.7503
O  core Fe core  -0.0555   7.9897  -0.3500  15.0000  -0.3000  36.0000
Ti core O  core  -0.1654   6.4224  -0.2493  16.1482  -0.3000  36.0000
Ti core Ti core  -0.0885   5.0000  -0.1924  14.9725  -0.2000  16.0000
reaxff2_bo
Fe core Fe core  -0.0771   6.4477  -0.2000  15.0000  -0.2000  16.0000
reaxff2_bond kcal
O  core O  core 142.2858 145.0000  50.8293  -0.2605   0.3451
O core Fe core  113.4349    0.0000    0.0000    0.1366    0.9495 # modify: 1
Fe core Fe core  38.7471   0.0000   0.0000   0.3595   1.0000
Ti core O core  100.3695   27.2153    0.0000    0.5110   -0.0707 # modify: 1 2
Ti core Ti core  80.1930   0.0000   0.0000  -0.8469   0.7528
reaxff2_over
O  core O  core   0.6051
O  core Fe core   0.0494
Fe core Fe core   0.2749
Ti core O  core   0.0100
Ti core Ti core   0.2022
reaxff2_morse kcal
O core Fe core    0.0846   10.0808    1.4284    1.7718   -1.0000   -1.0000 # modify: 4
Ti core O core    0.1200   10.5000    1.8000    1.9486    1.3476   -1.0000 # modify: 4 5
#
#  Angle parameters
#
reaxff3_angle kcal
```

```
O  core O  core O  core 80.7324 30.4554  0.9953  1.0783  1.6310
Fe core O  core O  core 79.7335  0.0100  0.1392  2.1948  0.4968
O  core Fe core Fe core 57.6787  4.8566  2.5768  1.0000  0.7552
O  core O  core Fe core 73.6721 32.6330  1.7223  1.4351  1.0221
Fe core O  core Fe core 65.7545  5.6268  4.0645  2.6730  1.7794
O  core Ti core O  core 86.0504 40.0000  2.6582  1.4546  0.4104
Ti core O  core Ti core 90.0000  7.9508  4.6213  1.7772  2.0000
O  core O  core Ti core 60.1752 17.8642  0.5300  1.1839  0.7799
O  core Ti core Ti core 14.8518 35.2791  0.9053  2.2837  2.0000
reaxff3_penalty kcal
O  core O  core O  core 50.0000
Fe core O  core O  core  0.0000
O  core Fe core Fe core  0.0000
O  core O  core Fe core  0.0000
Fe core O  core Fe core  0.0000
O  core Ti core O  core  0.0000
Ti core O  core Ti core  0.0000
O  core O  core Ti core  0.0000
O  core Ti core Ti core  0.0000
#
#  Torsion parameters
#
reaxff4_torsion kcal
O  core O  core O  core O  core  -2.5000 -25.0000  1.0000  -2.5000  -1.0000
X  core O  core O  core X  core   0.5511  25.4150  1.1330  -5.1903  -1.0000
```

2 Sensitivity of the parameters to tweaking

One can expect that some of the parameters will be more, and some less sensitive for tweaking. In order to justify the selection of the parameter set used in the work, we have carried out a series of parameters optimizations, with the final set of parameters plotted as the point on a scale from 70% to 130% of the initial value (a hard limit set in the procedure).

It can be observed that the distribution of the parameter values differs for different parameters. For instance – parameter 1 value converges at approximately 110% of the initial value, while parameter 5 is uniformly spread across the whole range. As in our procedure several parameters could be tweaked at once, some of the new values had positive, some negative effect, and some – had no effect at all. If the effect was negative – the set of parameters was rejected by the algorithm. On the other hand – if a tweaked parameter improved the match between the forces, some parameters which had no effect on the quality might have been accepted as well. This is represented in the Figure S1 ad the uniform spread across the whole assumed range of the values. We have concluded, that tweaking of these parameters was not necessary.

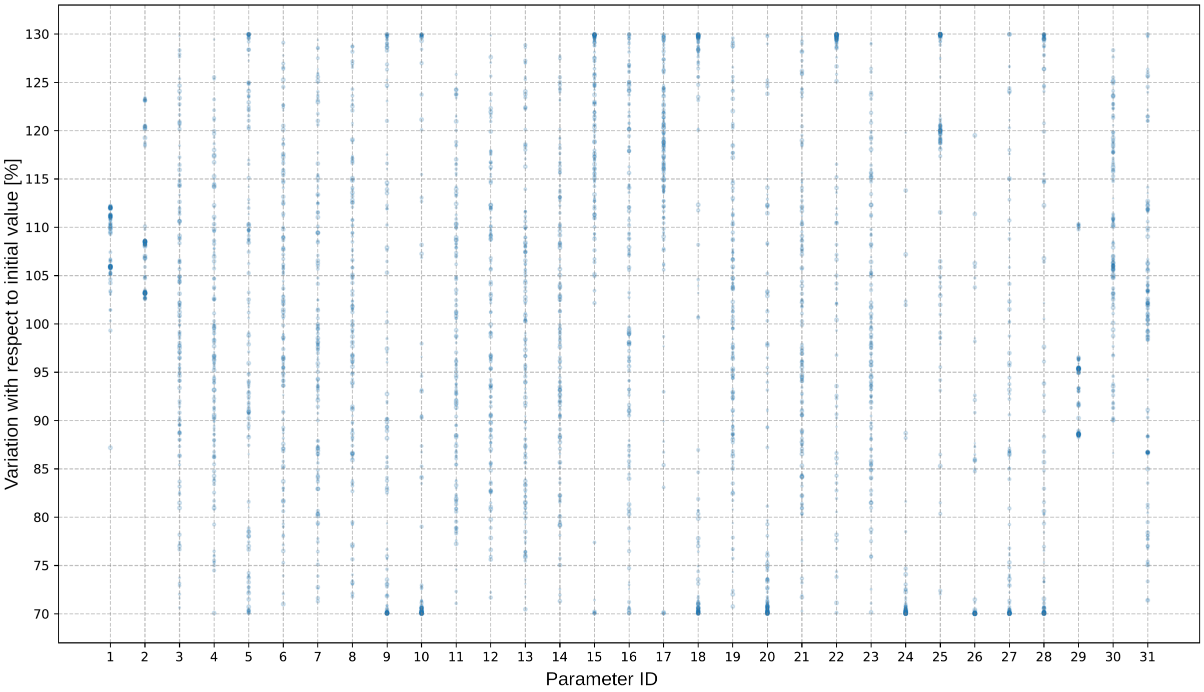

Figure 1: Sensitivity of the parameters to tweaking

Description:

parameter number	meaning	parameter number	meaning
1	O radius	17	Ti Morse pot.
2	O radius	18	Ti Morse pot.
3	Fe radius	19	Fe chi
4	Fe radius	20	Ti chi
5	Ti radius	21	Fe mu
6	Ti radius	22	Ti mu
7	Fe overcoordination	23	Fe gamma
8	Fe overcoordination	24	Ti gamma
9	Ti overcoordination	25	Fe-O bond energy
10	Ti overcoordination	26	Fe-O bond energy
11	Fe Morse pot.	27	Ti-O bond energy
12	Fe Morse pot.	28	Ti-O bond energy
13	Fe Morse pot.	29	Fe-O Morse pot.
14	Fe Morse pot.	30	Ti-O Morse pot.
15	Ti Morse pot.	31	Ti-O Morse pot.
16	Ti Morse pot.		
